# XRCC1 Arg194Trp and Arg280His Polymorphisms Increase Bladder Cancer Risk in Asian Population: Evidence from a Meta-Analysis

**DOI:** 10.1371/journal.pone.0064001

**Published:** 2013-05-21

**Authors:** Zhenqiang Fang, Fanglin Chen, Xiangwei Wang, Shanhong Yi, Wei Chen, Gang Ye

**Affiliations:** 1 Department of Urology, Center of Nephrology, The Second Affiliated Hospital of the Third Military Medical University, Chongqing, China; 2 Cancer Institute of PLA, The Second Affiliated Hospital of the Third Military Medical University, Chongqing, China; MOE Key Laboratory of Environment and Health, School of Public Health, Tongji Medical College, Huazhong University of Science and Technology, China

## Abstract

**Background:**

A lot of studies have investigated the correlation between x-ray cross complementing group 1 (XRCC1) polymorphisms and bladder cancer risk, but the results in Asian population were still inconclusive. We conducted a meta-analysis to ascertain the association of XRCC1 Arg194Trp, Arg280His and Arg399Gln polymorphisms with bladder cancer risk in Asian population.

**Methodology/Principal findings:**

The association strength was measured with odds ratios (ORs) and 95% confidence intervals (95% CIs). A total of 9 eligible studies, conducted in China, India and Japan, were identified. We observed a significant increased risk of bladder cancer in dominant model (OR = 1.199, 95% CI: 1.021,1.408, P_heterogeneity_ = 0.372), allele comparison (OR = 1.200, 95% CI: 1.057,1.362, P_heterogeneity_ = 0.107) of Arg194Trp, heterozygote comparison (OR = 1.869, 95% CI: 1.205,2.898, P_heterogeneity_ = 0.011) and dominant model (OR = 1.748, 95% CI: 1.054,2.900, P_heterogeneity_ = 0.01) of Arg280His. Pooled results estimated from adjusted ORs further validated these findings. No publication bias was detected. Subgroup analyses found that significant increased risk was only found among community-based studies not hospital-based studies. There was no evidence of publication bias.

**Conclusion:**

This is the first meta-analysis conducted in Asian investigating the correlation between XRCC1 polymorphisms and susceptibility to bladder cancer. Our meta-analysis shows that XRCC1 Arg194Trp and Arg280His polymorphisms are associated with a significantly increased risk of bladder cancer in Asian population.

## Introduction

Urinary bladder cancer is the sixth most common cancer, accounting for 14880 cases in the United States in 2012 [1], and the tenth in China [2]. Tobacco smoking and occupational exposure to certain chemical carcinogens have been established as the major risk factors of bladder cancer [3]. These carcinogens can cause DNA damage [4], and unrepaired DNA damages will lead to mutations and ultimately cancers [5]. Thus, impaired DNA repair capacity may alter susceptibility to cancers [6]. Epidemiological studies have showed that functional single nucleotide polymorphisms (SNPs) occurred in DNA repair genes are associated with cancer risk [7], [8].

Polymorphisms of x-ray cross complementing group 1 (XRCC1), a gene involved in the DNA base excision repair (BER) pathway, have been suspected with bladder cancer risk for decades. To date, hundreds of SNPs of XRCC1 have been validated and three of them were most extensively investigated: Arg194Trp in Exon 6 (rs1799782), Arg280His in Exon 9 (rs25489), and Arg399Gln in Exon 10 (rs25487). Numerous genetic association studies have investigated the correlation between XRCC1 polymorphisms and bladder cancer risk; however, the results were inconclusive or even contradictory. In 2008, two meta-analyses [9], [10] were conducted to determine the association, but no significant association was found. Notably, most studies included in the two meta-analysis were about Caucasians, and only one study [11] of Asians was included in Lao’s meta-analysis [9] and none in Wang’s meta-analysis [10]. Therefore, results of the two meta-analyses could not simply translate to Asian population. In addition, several recent genetic association studies [12]–[14] based on Asian population revealed significant association of XRCC1 Arg194Trp and Arg280His polymorphisms with bladder cancer risk. Although the sample size of these studies was small, they indicated that the association between XRCC1 polymorphisms and bladder cancer risk in Asian could be different from that in Caucasian. And a meta-analysis [15] also suggested that XRCC1 Arg194Trp polymorphism is a cancer susceptible factor among Chinese.

Therefore, it is necessary to perform a quantitative meta-analysis to answer the question whether XRCC1 polymorphisms are associated with bladder cancer risk in Asian. The present study is the first meta-analysis aimed to ascertain the correlation between XRCC1 polymorphisms (Arg194Trp, Arg280His, and Arg399Gln) and susceptibility to bladder cancer in Asian population.

## Methods

### Searching Strategy

Relevant studies were identified by searching databases of PubMed, EMBASE, and China National Knowledge Infrastructure (CNKI). The key words used for searching were as follows: “X-ray repair cross complementing group 1”, “single nucleotide polymorphism”, and “urinary bladder cancer”. Alternative spellings of these key words were also used and there was no limitation on languages or publication time. The last search was performed on December 14, 2012. References of previous meta-analyses were also manual searched to retrieve more studies.

### Inclusion and Exclusion Criteria

Studies met the following criteria were included: 1) a case-control study design; 2) investigating XRCC1 polymorphisms (Arg194Trp, Arg280His, and Arg399Gln) and bladder cancer risk; 3) full-text published articles; 4) detailed genotype data; 5) Asian population. For reports from the same study or center, the most recent one or the one with most participants was included. Two authors (Fang and Chen) selected eligible studies according to the inclusion criteria and reached consensus on each record.

### Data Extraction

Two authors (Fang and Chen) extracted data of eligible studies independently with a pre-designed data-collection form. The following data was collected: name of the first author, year of publication, country, source of control, SNPs investigated, number of cases and controls, genotype frequency in cases and controls, adjusted odds ratios (ORs) and 95% confidence intervals (95% CI). According source of control, eligible studies were categorized as hospital-based (HB) and community-based (CB). The two authors reached consensus on each item.

### Methodological Quality Assessment

Methodological quality of eligible studies was assessed using a quality scale (see Table S2 Methodological quality assessment scale) modified from previous studies [16], [17]. The quality scale consists of six items, namely, representativeness of cases, source of controls, ascertainment of cancer, sample size, quality control of genotyping, and Hardy-Winberg equilibrium (HWE). The quality score ranges from 0 to 10 and a high score indicates good quality.

### Statistical Analysis

The association strength of XRCC1 polymorphism with bladder cancer risk was measured with ORs and 95% CIs, and a 95% CI without 1 for OR indicated an increased or decreased risk of bladder cancer. The pooled ORs were calculated with fixed-effects model (Mantel-Haenszel method) or random-effects model (DerSimonian-Laird method). In the absence of significant heterogeneity, Mantel-Haenszel method based fixed-effects model was used; otherwise, random-effects model was used. Heterogeneity between studies was tested by chi-square based Q test and a P value less than 0.1 indicated the existence of significant heterogeneity. Four comparison models were calculated for each polymorphism: M1 (homozygote comparison, AA vs. aa), M2 (heterozygote comparison, Aa vs. aa), M3 (dominant model, AA+Aa vs. aa), M4 (recessive model, AA vs. Aa+aa) and M5 (allele comparison: A vs. a) (A: mutant allele, a: wild allele; 194Trp, 280His and 399Gln were considered as the mutant alleles). In the study reported by Wen et al [18], only M3 was calculated in that combined genotype frequency was provided. Adjusted ORs and CIs of M1, M2, and M3 extracted from eligible studies were also pooled. HWE in the controls of each study was tested by chi-square test for goodness of fit and a P value less than 0.05 indicated disequilibrium of HWE.

Subgroup analyses were conducted to explore the impact of source of controls and sample size. Sensitivity analysis was also performed by deleting one study each time. To explore the source of heterogeneity, meta-regression was performed and a P value less than 0.05 indicated a significant association. Publication bias was detected with Begg’s test and the Egger’s test, and a p<0.05 was considered significant [19]. All statistical analyses were calculated with STATA software (version 10.0; StataCorp, College Station, Texas USA). All P values are two-side.

## Results

### Characteristics of Eligible Studies

**Figure 1:**
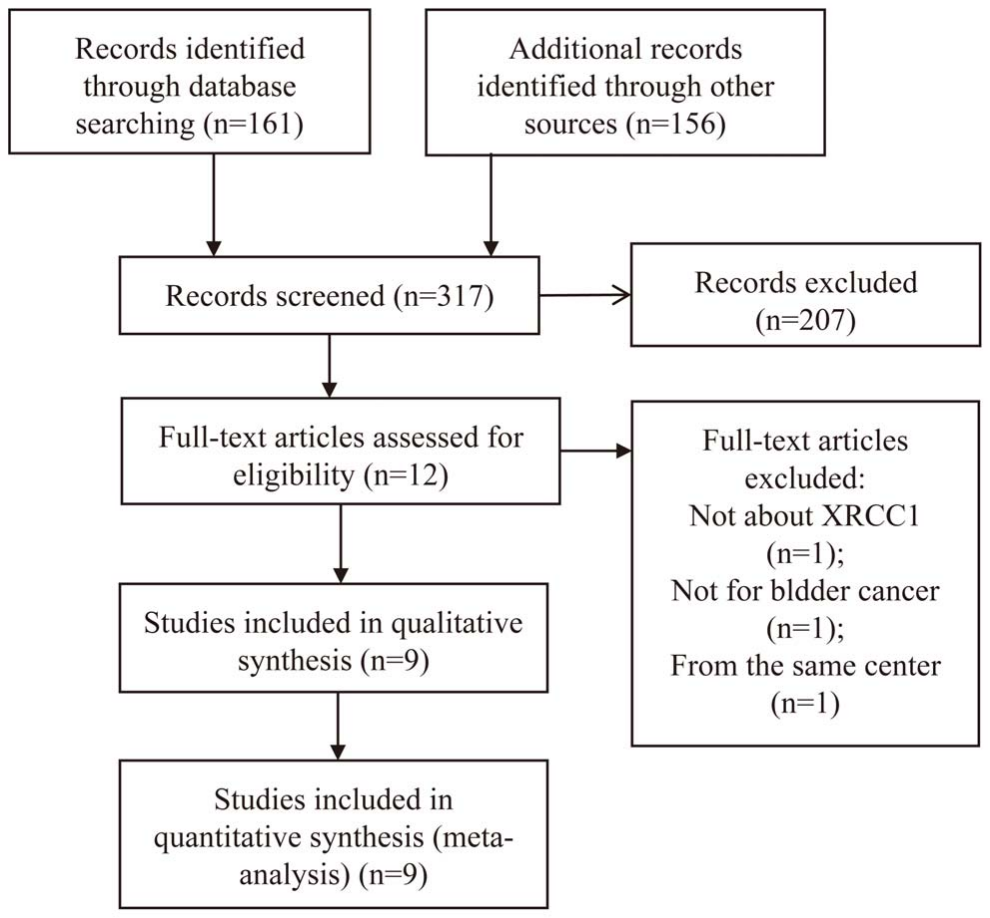
Flow Chart.

A number of 9 eligible studies [11]–[14], [18], [20]–[23], including 1931 cases and 2192 controls, were retrieved and the detail process was shown in Figure 1. After screening titles and abstracts, 12 full-text articles [11]–[14], [18], [20]–[23] were further reviewed for eligibility and 3 of them were excluded [24]–[26] (Figure 1). For studies of Mittal RD which were carried in the same place (Luchnow, India) [12], [24], the most recent one was included [12]. Eligible studies were conducted in China, India (Lucknow and other adjoining cities of North India), and Japan. A number of 6 studies [12], [13], [20]–[23] were about XRCC1 Arg194Trp polymorphism, 4 studies about Arg280His [12], [13], [20], [23], and 7 studies about Arg399Gln [11]–[14], [18], [20], [23] (Table 1).

**Table 1:** Characteristics of eligible studies.

Author	Year	Country	Source of control	No. CASE	No. CONTROL	Quality Score	Factors Adjusted
Zhi Y	2012	China	CB	302	311	6.5	age, gender, smoking
Mittal RD	2012	India	CB	212	250	5.5	age, smoking
Wang M	2010	China	HB	234	253	6.5	age, gender, smoking, alcohol use
Wen H	2009	China	HB	94	304	6.5	
Yang QX	2009	China	HB	220	220	6.5	
Hsu LI	2008	China	HB	221	223	7	age, gender, ethnicity
Arizono K	2008	Japan	HB	251	251	8.5	age, gender, smoking
Zhang W	2006	China	CB	242	225	6.5	age, smoking
Wu W	2006	China	HB	155	155	6.5	age, gender

CB: community-based studies; HB: hospital-based studies.

### Methodological Quality Assessment

Quality of included studies was acceptable with an average score of 6.6. Most studies had a small sample size and only 2 studies enrolled more than 500 participants [11], [14]. Deviation from Hardy-Winberg equilibrium in the controls was observed in 1 study [22] for Arg194Trp and 1 study [12] for Arg280His.

### Meta-analysis Results

**Figure 2:**
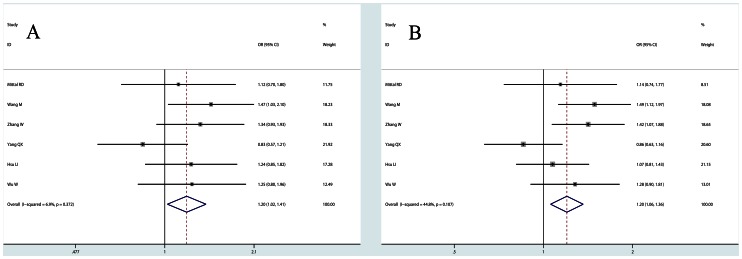
Forest plots for XRCC1 Arg194Trp polymorphism. A: dominant model: ArgTrp+TrpTrp vs. ArgArg; B: allele comparison: Trp vs. Arg.

*XRCC1 Arg194Trp* In pooled analysis, a significantly increased risk of bladder cancer was observed in dominant model (OR = 1.199, 95% CI: 1.021,1.408, P_heterogeneity_ = 0.372 Figure 2A) and allele comparison (OR = 1.200, 95% CI: 1.057,1.362, P_heterogeneity_ = 0.107 Figure 2B). By pooling adjusted ORs, we found that individuals with variant Trp194Trp genotype had an increased risk compared with those with wild Arg194Arg genotype (OR = 2.193, 95% CI: 1.099,4.376, P_heterogeneity_ = 0.019, Table 2). Both pooled results estimated by raw genotype distribution and adjusted ORs showed that carriers of the 194Trp allele were associated with high susceptibility to bladder cancer.

**Table 2:** Meta-analysis results of XRCC1 polymorphisms and bladder cancer risk.

	Arg194Trp			Arg280His			Arg399Gln		
	Study	OR(95% CI)	P	Study	OR(95% CI)	P	Study	OR(95% CI)	P
M1	[12], [13], [20]–[23]	1.729(0.964,3.101)	0.024	[12], [13], [20], [23]	2.113(0.565,7.901)	0.041	[11]–[14], [20], [23]	0.771(0.381,1.563)	0.001
M2	[12], [13], [20]–[23]	1.142(0.966,1.349)	0.635	[12], [13], [20], [23]	1.869(1.205,2.898) *	0.011	[11]–[14], [20], [23]	0.978(0.752,1.278)	0.018
M3	[12], [13], [20]–[23]	1.199(1.021,1.408)*	0.372	[12], [13], [20], [23]	1.748(1.054,2.900)*	0.01	[11]–[14], [18], [20], [23]	0.928(0.698,1.235)	0.001
M4	[12], [13], [20]–[23]	1.613(0.908,2.866)	0.021	[12], [13], [20], [23]	1.75(0.494,6.235)	0.052	[11]–[14], [20], [23]	0.788(0.437,1.422)	0.001
M5	[12], [13], [20]–[23]	1.200(1.057,1.362)*	0.107	[12], [13], [20], [23]	1.571(0.937,2.633)*	p<0.001	[11]–[14], [20], [23]	0.910(0.679,1.219)	p<0.001
M1a	[12], [13], [20], [22], [23]	2.193(1.099,4.376)*	0.019	[20], [23]	0.942(0.597,1.487)	0.978	[11]–[14], [20], [23]	0.783(0.387,1.582)	p<0.001
M2a	[12], [13], [20], [22], [23]	1.199(0.992,1.499)	0.954	[12], [13], [20], [23]	1.981(1.233,3.185) *	0.006	[11]–[14], [20], [23]	0.967(0.729,1.282)	0.011
M3a	[12], [20], [23]	1.241(0.982,1.567)	0.813	[20], [23]	1.950(0.923,4.118)	0.02	[11], [12], [14], [18], [20], [23]	1.041(0.702,1.545)	p<0.001

M1: homozygote comparison; M2: heterozygote comparison; M3: dominant model; M4: recessive model; M5: allele comparison; P: P value for heterogeneity; a estimated from adjusted ORs and 95% CIs; NA: not analyzed;

* significant difference.

**Figure 3:**
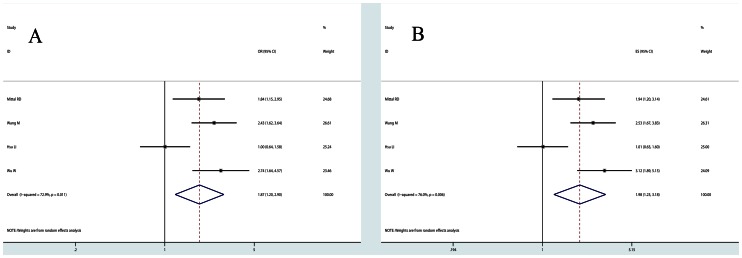
Forest plots XRCC1 Arg280His polymorphism. Heterozygote comparison (ArgHis vs. ArgArg) estimated with raw genotype frequencies (A) and adjusted odds ratios (B).

*XRCC1 Arg280His* Dominant model comparison suggested that carriers of the 280His allele are associated with increased risk (OR = 1.748, 95% CI: 1.054,2.900, P_heterogeneity_ = 0.01). The variant heterozygote genotype of Arg280His increased bladder cancer risk compared with wild homozygote (OR = 1.869, 95% CI: 1.205,2.898, P_heterogeneity_ = 0.011, Figure 3A). This association was further validated by the pooled results estimated from adjusted ORs (Heterozygote comparison, OR = 1.981, 95% CI: 1.233,3.185, P_heterogeneity_ = 0.006, Figure 3B) (Table 2).

*XRCC1 Arg399Gln* As for *Arg399Gln* polymorphism, we did not found any evidences of significant association in any comparison (Table 2). Notably, heterogeneity was significant in most comparison models.

### Meta-regression and Subgroup Analyses

To detect the source of heterogeneity, meta-regression was performed for the Arg399Gln (all 7 studies were included in this comparison model). We found that source of controls (P = 0.018 in dominant model) was responsible for heterogeneity but not sample size (P = 0.886 in dominant model).

Subgroup analyses also confirmed that heterogeneity was caused by sources of control in that heterogeneity was only significant in the subgroup of HB studies but not CB studies (Table 3). Due to limited number of studies, subgroup analyses were only performed for sources of control. As Table 3 shows, increased risk was found only among CB studies but not HB studies.

**Table 3:** Subgroup analyses of XRCC1 Arg194Trp and Arg399Gln polymorphisms and bladder cancer risk.

		Community						Hospital				
		Study		OR(95% CI)		P		Study	OR(95% CI)		P	
XRCC1 Arg399Gln												
M1	[12], [22]		1.651(1.101,2.478) *		0.655		[13], [20], [21], [23]			0.506(0.209,1.230)		0.001
M2	[12], [22]		1.364(1.054,1.764) *		0.611		[13], [20], [21], [23]			0.823(0.642,1.057)		0.204
M3	[12], [22]		1.411(1.104,1.805) *		0.714		[13], [20], [21], [23]			0.777(0.593,1.017)		0.065
M4	[12], [22]		1.363(0.937,1.985)		0.482		[13], [20], [21], [23]			0.569(0.252,1.284)		0.02
M5	[12], [22]		1.286(1.076,1.536)*		0.984		[13], [20], [21], [23]			0.760(0.679,1.219)		0.002
XRCC1 Arg194Trp												
M1	[12], [14]		2.952(1.422,6.127)*		0.558		[11], [13], [20], [23]			1.407(0.722,2.994)		0.025
M2	[12], [14]		1.38(0.844,1.535)		0.811		[11], [13], [20], [23]			1.143(0.935,1.398)		0.339
M3	[12], [14]		1.256(0.940,1.676)		0.552		[11], [13], [18], [20], [23]			1.175(0.969,1.425)		0.181
M4	[12], [14]		2.774(1.359,5.664)*		0.598		[11], [13], [20], [23]			1.366(0.688,2.710)		0.026
M5	[12], [14]		1.330(1.049,1.686)*		0.419		[11], [13], [20], [23]			1.151(0.991,1.362)		0.06

M1: homozygote comparison; M2: heterozygote comparison; M3: dominant model; M4: recessive model; M5: allele comparison; P: P value for heterogeneity; a estimated from adjusted ORs and 95% CIs;

* significant difference.

### Publication Bias and Sensitivity Analyses

**Figure 4:**
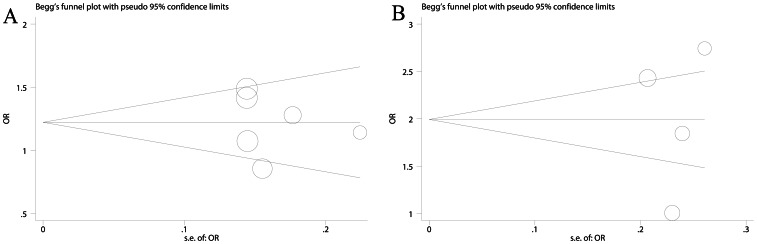
Funnel plots for XRCC1 Arg194Trp (A, allele comparison: Trp vs. Arg) and Arg280His (B, heterozygote comparison: ArgHis vs. ArgArg).

Egger’s test and Begg’s test were conducted for the three polymorphisms. Briefly, no evidence of significant publication bias was detected. For example, Begg’s funnel plots were roughly symmetrical for allele comparison of XRCC1 Arg194Trp and heterozygote comparison of Arg280His (Figure 4) and quantitative tests showed that no publication bias existed (Arg194Trp: P_Begg_ = 0.452, P_Egger_ = 0.710; Arg399Gln: P_Begg_ = 0.734, P_Egger_ = 0.921). Sensitivity analyses, which assess the impact of individual study on pooled results, revealed that no individual study affected pooled results significantly (data not shown). These results suggested that results of our meta-analyses were reliable and robust.

## Discussion

XRCC1 is an important member of BER pathway, which repairs single-strand breaks, and XRCC1 is crucial to the integrity of chromosome. The XRCC1 protein acts as a scaffold for other DNA repair proteins, like polynucleotide kinase, human AP endonuclease (APE1), DNA polymerase β, DNA ligase III, and poly(ADP-ribose) polymerases (PARP) [27]. The three functional SNPs (Arg194Trp, Arg280His, and Arg399Gln) can cause amino acid substitutions. Arg194Trp and Arg280His polymorphisms locate at the linker region connecting the domains that interact with PARP and DNA polymerase β, while Arg399Gln resides in PARP-binding domain [28]. These function SNPs lead to altered DNA damage repair capacity. For example, the variant XRCC1 194Trp allele was associated with an increase in DNA strand breaks after exposure to bleomcyin [29] and XRCC1 Arg399Gln polymorphism was associated with higher level of sister chromatid exchange and DNA adducts [30]. Thus, it is reasonable to conclude that functional SNPs of XRCC1 are associated with susceptibility to bladder cancer.

Polymorphisms of XRCC1 and bladder cancer risk have been investigated by many studies, most of which focused on Caucasian, and no significant association of XRCC1 polymorphisms with bladder cancer risk was found. However, evidence in Asian population was limited and the result was inconclusive. Thus we performed this meta-analysis to determine whether XRCC1 polymorphisms were associated with susceptibility to bladder cancer in Asian, and this is the first meta-analysis on this topic.

Different from previous meta-analyses [9], [10], we found that XRCC1 Arg194Trp and Arg280His polymorphisms significantly increased bladder cancer risk in Asian population. The Arg194Trp polymorphism led to an increased risk in dominant model and allele comparison (Table 2), which was in consistent with a previous meta-analysis among Chinese [15]. On the other hand, the heterozygote comparison and dominant model revealed that the Arg280His polymorphism increased susceptibility to bladder cancer (Table 2). Smoking is a validated risk factor for bladder cancer, and we collected ORs, which were adjusted for smoking habit, from included studies. By pooling adjusted ORs, Arg194Trp and Arg280His polymorphisms were still associated with increased risk, which further confirmed our findings. Results of the present study suggested that study design would affect the association of bladder cancer risk, since we found, in both the comparisons of Arg194Trp and Arg280His, that CB studies revealed an increased risk but HB studies suggested no significant association. The differences between CB and HB studies showed that selection bias might existed in HB studies. In addition, subgroup analyses showed that heterogeneity was only found among HB studies.

Limitation of this meta-analysis should also be noted. First, our results were based on studies with small sample size, and the number of studies for each polymorphism was also small, which might lead to a small study effect. Second, for Arg280His, only 4 studies [12], [13], [20], [23] were available and 1 [12] of them were deviated from HWE. Given the limited number of studies, we did not conduct further subgroup analyses; however, Egger’s test, Begg’s test and sensitivity analyses showed this association was reliable and robust.

The association revealed by our meta-analysis is different from that in Caucasian population [9], [10], which suggests the difference between Asians and Caucasians. The difference could be explained by genetic background, different risk factors in life styles, and the exposure to different environmental factors. According to Lao’s meta-analysis, the 194Arg allele frequency was 93.5% in controls of Caucasian, but according to our results, the 194Arg frequency was significantly lower in controls of Asian (77.4%, p<0.01). This suggests that the different associations between Caucasian and Asian may be partially attributed to different genetic background. Thus, results of genetic association studies could not simply translate to another ethnicity. Our meta-analysis also suggests that study design is critically important for genetic association studies in that significant association was only found among CB studies not HB studies in this meta-analysis.

In this meta-analysis, we also found that XRCC1 Arg194Trp was significantly associated with increased bladder cancer risk in that the OR values were larger than 2 (Table 2). Given that the predictive value of XRCC1 polymorphisms on platinum-based chemotherapy in non-small cell lung cancer has already been validated, such as XRCC1 Arg194Trp [31], it is reasonable to conclude that Arg194Trp may play a similar role in bladder cancer. The potential value as a biomarker for Arg194Trp in bladder cancer warranted further investigation. Furthermore, a novel polymorphism in the promoter region of XRCC1 (−77T>C, rs3213245) has been identified recently [32], [33]. Liu and colleagues suggested that the −77T>C polymorphism was associated with breast cancer risk [34] but this polymorphism did not predict clinical outcomes of platinum-based chemotherapy of patients with non-small cell lung cancer [35]. Further studies are warranted clarify the exact role of XRCC1 −77T>C. It has been demonstrated that XRCC1 polymorphisms could increase cancer risk by interacting with other gene polymorphisms in a multiplicative manner, such as adenosine diphosphate ribosyl transferase Val762Ala and XRCC1 Arg399Gln polymorphisms [36]. Thus, it is necessary to consider the effect of gene-interaction in further genetic association studies.

In summary, this is the first meta-analysis investigating the correlation between XRCC1 polymorphisms and bladder cancer risk in Asian and our results suggest that XRCC1 Arg194Trp and Arg280His polymorphisms are associated with increased risk of bladder cancer in Asian population. Well-designed studies with large sample size are warranted to determine the role of XRCC1 polymorphisms in bladder cancer, especially for Arg194Trp and Arg280His.

## Supporting Information

Table S1 **PRISMA checklist.**
(DOC)

Table S2 **Methodological quality assessment scale.**
(DOC)
